# Intracellular localization of Saffold virus Leader (L) protein differs in Vero and HEp-2 cells

**DOI:** 10.1038/emi.2016.110

**Published:** 2016-10-12

**Authors:** Yishi Xu, Carla Bianca Luena Victorio, Qimei Ng, Mookkan Prabakaran, Yee-Joo Tan, Kaw Bing Chua

**Affiliations:** 1 Temasek Life Sciences Laboratory, 1 Research Link, National University of Singapore, Singapore 117604, Singapore; 2 Department of Microbiology and Immunology, Yong Loo Lin School of Medicine, National University Health System, National University of Singapore, Singapore 119228, Singapore; 3 Institute of Molecular and Cell Biology, A*STAR (Agency for Science, Technology and Research), Singapore 138673, Singapore

**Keywords:** leader protein, protein localization, Saffold virus, viral protein

## Abstract

The Saffold virus (SAFV) genome is translated as a single long polyprotein precursor and co-translationally cleaved to yield 12 separate viral proteins. Little is known about the activities of SAFV proteins although their homologs in other picornaviruses have already been described. To further support research on functions and activities of respective viral proteins, we investigated the spatio-temporal distribution of SAFV proteins in Vero and HEp-2 cells that had been either transfected with plasmids that express individual viral proteins or infected with live SAFV. Our results revealed that, with the exception of the Leader (L) protein, all viral proteins were localized in the cytoplasm at all the time points assayed. The L protein was found in the cytoplasm at an early time point but was subsequently translocated to the nucleus of HEp-2, but not Vero, cells. This was observed in both transfected and infected cells. Further mutational analysis of L protein revealed that Threonine 58 of the Ser/Thr-rich domain of L protein is crucial for protein trafficking between the cytoplasm and nucleus in HEp-2 cells. These findings contribute to a deeper understanding and stimulate investigation of the differetial cellular responses of HEp-2 cells in comparison to other mammalian cell lines during SAFV infection.

## Introduction

Saffold virus (SAFV) belongs to the Theilovirus species in the Cardiovirus genus under the family Picornaviridae.^1, 2, 3^ First reported in 2007 by Jones *et al.*,^4^ SAFV is currently considered as the first human cardiovirus virus. SAFV infection is most probably widespread among young children worldwide based on the available published seroprevalence studies.^3, 5, 6^ SAFV is a non-enveloped single-stranded RNA virus of positive polarity. Its genome is comprised of 8050 nucleotides and, like other cardioviruses, has one open reading frame flanked by a long non-coding region at its 5′ end and a shorter non-translated region at its 3′ end. The single open reading frame is translated into a polyprotein precursor with 13 cleavage sites and co-translationally cleaved to yield 12 separate viral proteins: Leader (L), VP4, VP3, VP2, VP1, 2A, 2B, 2C, 3A, 3B, 3C, and 3D. The L protein is a 71 amino-acid long protein located at the N-terminal of the SAFV polyprotein^4^ and contains four domains: a zinc finger domain,^7^ an acidic central domain, a Ser/Thr-rich domain, and a Theilo domain.^8^ The L protein of SAFV and Theiler's murine encephalitis virus (TMEV)—the phylogenetically closest cardiovirus relative of SAFV—share 62% similarity in sequence. Currently, the associated functions of L protein of SAFV include interfering with the transcription of immediate-early alpha/beta interferon,^9^ inducing the phosphorylation of Phe/Gly-containing nucleoporins^10^ and inhibiting stress granule assembly.^11^

Previous studies on TMEV and other cardioviruses revealed that knowledge of cellular localization of viral proteins might serve as pointers for the discovery of their respective interacting partner protein(s) and the subsequent elucidation of their functions.^4^ Though of the same virus type, the GDVII and DA strains of TMEV had been demonstrated to induce divergent host infection phenotypic characteristics; the neurovirulent GDVII strain was shown to cause acute and fatal encephalomyelitis in mice without leading to persistent central nervous system infection, whereas the DA strain causes mild induced encephalomyelitis followed by viral persistence in the central nervous system that resulted in a chronic inflammatory demyelinating disease of the infected mice.^12^ Moreover, the L protein of TMEV was reported to localize either^1^ in both the nucleus and cytoplasm (DA strain) or^2^ the cytoplasm only (GDVII strain).^13^ The L protein of DA strain was known to interfere with the nucleocytoplasmic trafficking of cellular proteins; it promotes the redistribution of a nuclear protein to the cytoplasm and a cytoplasmic protein to the nucleus.^14^ It was also demonstrated to inhibit the production of immediate-early alpha/beta interferon at the transcription level,^15^ which led to the dysregulation of the whole cellular defense system by probably fostering the persistence of TMEV DA strain. These results supported the idea that knowledge of cellular localization of SAFV proteins, especially the L protein, might provide future directions for exploring the functions and activities of these respective viral proteins in infected host cell phenotypes. However, the molecular and cellular study of the viral proteins of SAFV, including their cellular localization, had been extremely limited owing to the arduousness of culturing SAFV *in vitro*.^1^

Our previous work on SAFV-Penang revealed that the virus could induce various mammalian cell lines to undergo apoptosis, but the apoptotic activity in infected HEp-2 cells failed to proceed to the end point.^16^ This phenomenon suggested that the infection of SAFV or activity of its viral proteins in HEp-2 might be different from other cell lines tested, and knowing the cellular localization of SAFV proteins, however, could provide clues on the underlying reason why SAFV-infected HEp-2 cells fail to undergo full apoptosis. In this study, we investigated the cellular localization of individual SAFV proteins, including L, 1D, 2A, 2B, 2C, 3A, 3C and 3D, in HEp-2 and Vero cells. We focused specifically on the L protein.

## Materials and Methods

### CELLS AND VIRUSES

The cell lines used in this study were originally derived from human laryngeal carcinoma sample (HEp-2, CCL-23), African green monkey kidneys (Vero, CCL-81), mouse neurons (Neuro2A, CCL-131)), mouse fibroblasts (NIH/3T3, CRL-1658)) and hamster kidneys (CHO-K1, CCL-61), which were obtained from American Type Culture Collection (ATCC, Manassas, VA, USA). These cells were grown in Dulbecco's modified Eagle's medium (DMEM, Gibco, Grand Island, NY, USA) supplemented with 10% fetal bovine serum (FBS, i-DNA, Singapore, Singapore) and 0.22% (w/v) sodium bicarbonate (NaHCO_3_, Sigma-Aldrich, St Louis, MO, USA) and incubated at 37 °C in 5% CO_2_. The origin and passage history of Saffold virus (SAFV-Penang strain, Genbank number: HQ162476) used in this study have been described.^5^

### REAGENTS

The following reagents and antibodies were purchased commercially: mouse anti-Myc (Santa Cruz Biotechnology Inc., Santa Cruz, CA, USA); goat anti-Ran GTPase (Santa Cruz Biotechnology Inc.); rabbit anti-tubulin (Sigma-Aldrich); rabbit anti-β-actin (Cell Signaling Technology, Beverly, MA, USA); rabbit anti-mouse immunoglobulins–horseradish peroxidase (IgG-HRP) (Dako, Glostrup, Denmark); swine anti-rabbit IgG-HRP (Dako); rabbit anti-goat IgG-HRP (Dako, Glostrup, Denmark); mouse anti-polyhistidine (Sigma-Aldrich); swine anti-rabbit immunoglobulins-fluorescein isothiocyanate (IgG-FITC) (Dako); rabbit anti-mouse IgG-FITC (Dako); clarity enhanced chemiluminescence solution (Bio-Rad, Hercules, CA, USA); and Hoechst 33258 (Molecular Probes, Invitrogen Corp., Carlsbad, CA, USA).

### PLASMID CONSTRUCTS

The plasmids for the expression of viral proteins in mammalian cells were constructed in the pXJ40-Myc vector (the construction of this vector had been described elsewhere),^17^ in which gene transcription is under the control of the human cytomegalovirus promoter. The complementary genomic DNA (cDNA) of SAFV was used to serve as templates for PCR amplification of the genes encoding viral proteins and primer-mediated mutated L proteins. The primers used to obtain the respective viral gene fragments and mutated L gene fragments are listed in Supplementary Table S1. After digestion with appropriate restriction enzymes, the fragments were ligated into XhoI-PstI (L, mutated L, 2C and 3D), BamHI-PstI (1D and 2B) or BamHI-XhoI (2A, 3A and 3C) cloning site of pXJ40-Myc plasmids. The resultant plasmids containing the inserts for the expression of viral proteins were named as pXJ40-Myc-L, -1D, -2A, -2B, -2C, -3A, -3C or -3D. The plasmids carrying the inserts for the expression of mutated L proteins (zinc finger domain deleted L protein, acidic central domain deleted L protein, Ser/Thr-rich domain deleted L protein or Theilo domain deleted L protein) were named as pXJ40-Myc-LΔZ, -LΔA, -LΔS/T or -LΔC, respectively.

One-step site-directed mutagenesis was performed to generate constructs with Alanine substitutions in the Ser/Thr-rich domain and phosphorylation mimic on Thr 58 of L protein.^18^ Plasmid pXJ40-MyC-L served as the template for all the site-directed mutagenesis experiments. The primers used are listed in Supplementary Table S1.

The plasmids used as the expression vector of viral proteins in *Escherichia coli* strain M15 (pREP4) were constructed with the pQE30 vector (Qiagen, Valencia, CA, USA). The PCR products of L, 1D and 2C were amplified using the primers listed (Supplementary Table S1). After digestion with appropriate restriction enzymes, L and 2C were ligated into the SacI-SacI cloning site, while 1D was cloned into the SacI-KpnI site of pQE30 plasmids.

### POLYCLONAL ANTIBODY PRODUCTION

The rabbit anti-L, -1D and -2C polyclonal antibodies were produced ‘in-house'. The SAFV cDNA encoding L, 1D or 2C protein were cloned into the pQE-30 vector (Qiagen) and transformed into *E. coli* strain M15 (pREP4) competent cells for protein expression. The expression of hexahistidine-tagged fusion L, 1D or 2C was induced overnight by the addition of one mM isopropyl β-D-1-thiogalactopyranoside and purified on a Ni-NTA column (Qiagen).^19^ The efficiency of protein expression and purification were checked by sodium dodecyl sulfate–polyacrylamide gel electrophoresis (SDS-PAGE) and western blotting analysis.

The purified L, 1D or 2C protein was separately mixed with complete Freund's adjuvant in a 1:1 ratio and injected into two female New Zealand rabbits (0.4 mg/rabbit). Booster shots containing purified proteins mixed with incomplete Freund's adjuvant were performed 3–4 times at two weekly intervals (0.3 mg/rabbit). Rabbit antisera were collected 10 days after the final injection and tested for specificity by western blottings against the purified proteins and infected Vero cell lysates, as well as immunofluorescent staining of SAFV-infected Vero cell lysates. Polyclonal antibody production method was reviewed and approved by the Institutional Animal Care and Use Committee (IACUC) of the Temasek Life Sciences Laboratory, Singapore, Singapore (IACUC approval number TLL-047-12), following guidelines set by the National Advisory Committee for Laboratory Animal Research of Singapore.

### SDS-PAGE AND WESTERN BLOTTING ANALYSIS

To test the efficiency of SAFV L, 1D or 2C protein expression and purification, samples collected in each step of the process were used to perform SDS-PAGE and western blotting analysis. Samples (20 μg each) were electrophoresed on 16% or 14% SDS-polyacrylamide gels. The SDS-PAGE gels were either stained with Coomassie Blue (Bio-Rad, Philadelphia, PA, USA) (for SDS-PAGE analysis) or transferred onto polyvinylidene difluoride (PVDF) membranes (Bio-Rad) (for western blotting analysis). PVDF membranes were then blocked for one h at room temperature in a suspension of 5% (w/v) blotting grade non-fat milk dissolved in phosphate-buffered saline (PBS) supplemented with 1% Tween-20 (PBS-T), and incubated overnight at 4 °C with mouse anti-His antibody in PBS-T buffer supplemented with 5% non-fat milk. The membranes were washed three times with PBS-T and subsequently incubated at room temperature for one h with rabbit anti-mouse IgG-HRP in 5% (w/v) non-fat milk in PBS-T.

The purified proteins and cell lysates of SAFV-infected Vero cells were used for testing the antibody efficiency and specificity of rabbit polyclonal antibody. Protein samples (20 μg each) were electrophoresed on 16% SDS-polyacrylamide gels and transferred onto PVDF membranes. The subsequent steps were similar to those mentioned above. The primary and secondary antibodies used in this experiment were of different dilutions of rabbit polyclonal antibodies and swine anti-rabbit IgG-HRP, respectively.

To check the expression of viral proteins after transfection, the cells transfected with pXJ40-Myc-L, -1D, -2A, -2B, -2C, -3A, -3C, -3D, -LΔZ, -LΔA, -LΔS/T or -LΔC were harvested at 24 h posttransfection, or every four h after transfection in the case of 3C protein, and lysed with RIPA buffer (50 mM Tris·Cl, pH 8.0; 1% NP-40; 0.5% sodium deoxycholate; 150 mM NaCl; 1% SDS; protease inhibitor). Protein samples (20 μg each) were electrophoresed on 16% or 12% SDS-polyacrylamide gels and transferred onto PVDF membranes. The subsequent steps were similar to those mentioned above. The primary antibody used in the experiment was mouse anti-Myc antibody, and the secondary antibody was rabbit anti-mouse IgG-HRP antibody.

All the experiments involving SDS-PAGE and western blotting analysis were independently repeated three times.

### CELL FRACTIONATION

The cells transfected with pXJ40-MyC-L were harvested in lysis buffer (7.5 mM NaCl; 1 mM HEPES, pH 7.2; 0.02 mM EDTA, 1% Triton X-100; protease inhibitor) at 12, 24, 36 and 48 h posttransfection after washing with cold PBS twice. The cells were then incubated on ice for 30 min with shaking every five min, followed by centrifugation at 4 °C and 1000 *g* for 3 min. The resultant supernatant contains cytoplasmic protein, and pellet contains nuclear protein. Protein samples (160 μg each) were then electrophoresed on 16% SDS-polyacrylamide gels and transferred onto PVDF membranes. The subsequent steps were similar to other western blotting experiments mentioned above. The primary antibodies used in the experiment were mouse anti-Myc antibody, goat anti-Ran antibody and rabbit anti-tubulin antibody. The secondary antibodies were rabbit anti-mouse IgG-HRP antibody, rabbit anti-goat IgG-HRP antibody and swine anti-rabbit IgG-HRP antibody.

### TRANSFECTION

Cells seeded overnight in six-well-plate (4 × 10^5^ cells/well), 25 cm^2^ flask (1 × 10^6^ cells/well) or eight-well Lab-Tek chamber slides (1.5 × 10^4^ cells/well) (Nunc, Naperville, IL, USA) were transfected with reaction mixtures consisting of 2.5 μg (six-well plate) or 0.25 μg (eight-well Lab-Tek chamber slides) of DNA of the respective expression vectors and Lipofectamine 2000 (Invitrogen) according to the manufacturer's manual. Plates were incubated at 37 °C in 5% CO_2_ for the indicated times.

### VIRUS INFECTION

Cells were seeded overnight in eight-well Lab-Tek chamber slides (2 × 10^4^ cells/well) in DMEM (10% FBS). The cells were adapted to DMEM (1% FBS) for one h at 37 °C in 5% CO_2_ prior to incubation with the virus at a multiplicity of infection of 1. Cells were fixed at 12, 24, 36 or 48 h postinfection. The time of inoculation was designated at zero (0) time point.

### IMMUNOFLUORESCENCE MICROSCOPY

To verify the cellular localization of various SAFV viral proteins, both HEp-2 and Vero cells transfected with expression plasmids or infected with SAFV on eight-well Lab-Tek chamber slides were used. The cells were fixed with 4% paraformaldehyde for 15 min at room temperature. After permeabilization with PBS-1% Triton-X for 15 min at room temperature, cells were incubated with anti-Myc, -L, -1D or -2C antibody for one h at 37 °C. The cells were washed three times with PBS and subsequently incubated with anti-mouse-FITC (transfected cells) or anti-rabbit-FITC antibody (infected cells). Cell nuclei were stained with Hoechst 33258. Cells were observed with a fluorescence microscope (Leica SP8 laser scanning confocal microscope (Leica Microsystems CMS GmbH, Mannheim, Germany) with a 63 × /1.40 NA or 40 × /1.30 NA oil objective). Positively stained cells were classified into three types as shown in Supplementary Figure S1. HEp-2 cells transfected with pXJ40-Myc-L at 48 h posttransfection served as the reference for characterization. Type A cells had clear-cut positive immunofluorescence staining in the cytoplasm only, type B cells had positive staining in the cytoplasm with speckles of positive staining overlapping the nuclear region, and type C cells had clear-cut positive staining in both the cytoplasm and the nucleus. Approximately 100 immunofluorescent-positive cells from at least five different optical fields were randomly selected and evaluated in each experiment, and three independent experiments were performed. Intracellular viral protein localization was determined by scoring the ratio of the number of cells based on the nuclear/cytoplasmic profiles of the type of positive immunofluorescence to the total number of positive cells counted.

The SAFV-infected Vero cells were used for testing the antibody efficiency and specificity of rabbit polyclonal antibodies. The infected cells were fixed with 4% paraformaldehyde for 15 min before permeabilization with PBS-1% Triton-X for 15 min at room temperature. Cells were then incubated with various concentration of rabbit anti-L, 1D or 2C antibody for one h at 37 °C. The cells were washed three times with PBS and subsequently incubated with anti-rabbit IgG-FITC antibody (infected cells). Cell nuclei were stained with Hoechst 33258. Cells were observed with a fluorescence microscope (Leica SP8 laser scanning confocal microscope with a 63 × /1.40 NA oil objective). The experiments were repeated three times independently.

### STATISTICAL ANALYSIS

Chi-square test was used to verify the significance of the association between variables in this study. The differences between variables were considered statistically significant at *P*<0.01.

## Results

### VIRAL PROTEINS EXPRESSED IN TRANSFECTED HEP-2 AND VERO CELLS

**Figure 1:**
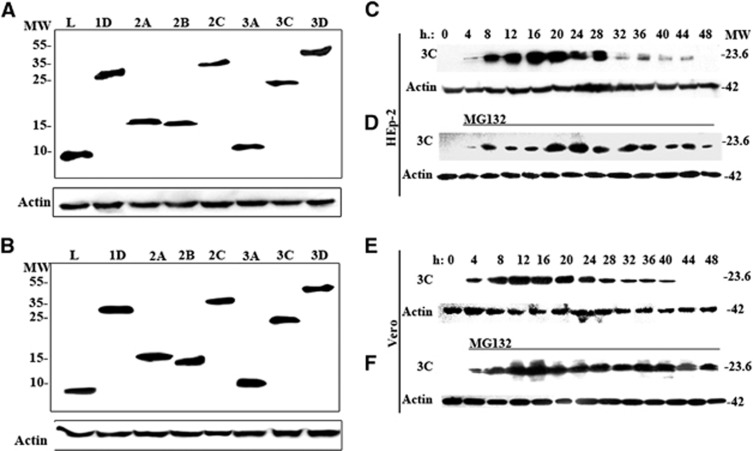
Protein expression profiles of HEp-2 and Vero cells transfected with pXJ40-Myc-SAFV virus gene constructs. (**A**, **B**) Western blottings of the expressed viral proteins in transfected (**A**) HEp-2 cells and (**B**) Vero cells at 24 h posttransfection. (**C**–**F**) Time-course study of the expression of 3C protein in transfected (**C**, **D**) HEp-2 and (**E**, **F**) Vero cells, (**C**, **E**) without treatment and (**D**, **F**) in the presence of 20 μM reversible 26S proteasome inhibitor MG132. The cell lysates of HEp-2 or Vero cells expressing 3C were collected every 4 h after transfection until 48 h posttransfection. The membranes were stained with anti-Myc or anti-β-actin antibody, and the intensity of β-actin staining served as the protein-loading control. This image is representative of three independent experiments. Lanes L, 1D, 2A, 2B, 2C, 3A, 3C and 3D represent the expression of the respective viral genes. molecular weight marker (in thousands Dalton), MW; Saffold virus, SAFV.

**Figure 2:**
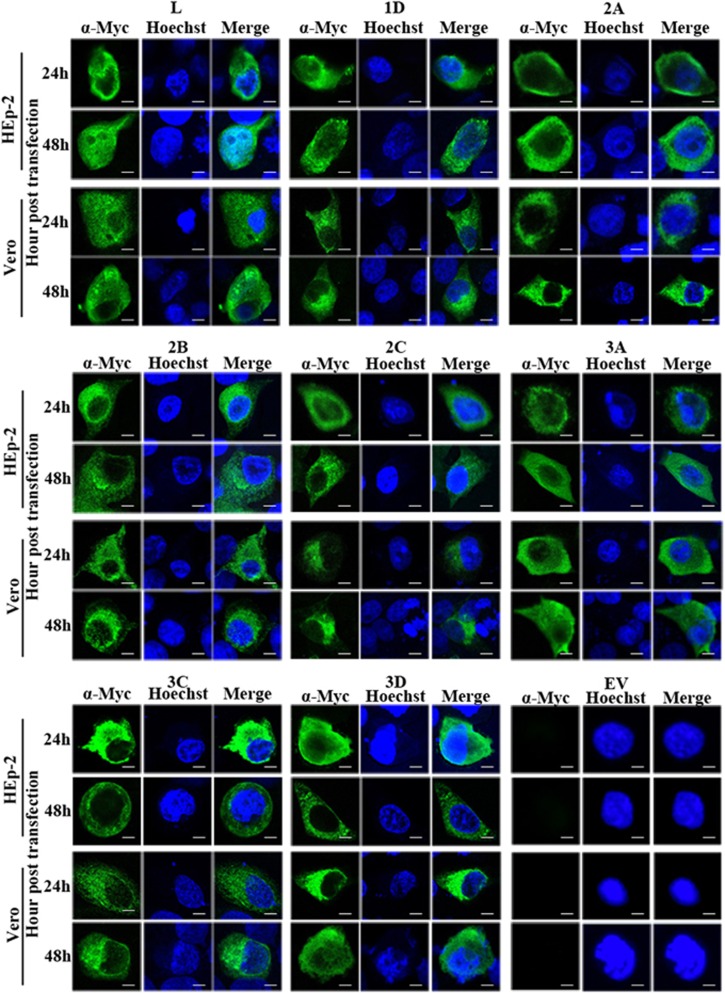
Cellular localization of viral proteins in transfected cells. Immunofluorescent detection of individual SAFV proteins—L, 1D, 2A, 2B, 2C, 3A, 3C and 3D—in transfected HEp-2 and Vero cells at 24 and 48 h posttransfection. HEp-2 and Vero cells were transfected with the expression plasmids pXJ40-Myc-SAFV virus gene constructs, fixed at 24 or 48 h posttransfection and immunofluorescently stained with the anti-Myc antibody. Cell nuclei were stained with Hoechst 33258. Cells were observed with a fluorescence microscope (Leica SP8 laser scanning confocal microscope with a 63 × /1.40 NA oil objective). Merge represents the merged images stained with anti-Myc and Hoechst. EV represents cells transfected with empty vector; Saffold virus, SAFV. Scale bar=10 μM.

The respective individual viral protein genes (L, 1D, 2A, 2B, 2C, 3A, 3C and 3D) of SAFV-Penang were cloned into the pXJ40-Myc vector, and the resulting vectors were separately transfected into HEp-2 and Vero cells. At 24 h posttransfection, the expression of respective Myc-tagged viral proteins was assessed by western blotting analysis. Figures 1A and 1B show the SAFV proteins at their expected molecular sizes. Similar profiles were observed in both cell lines. The expression of each viral protein at 24 and 48 h posttransfection was further examined by indirect immunofluorescence staining using anti-Myc antibody. In Figure 2, both HEp-2 and Vero cells transfected with vectors expressing each of the respective viral proteins showed strong fluorescence signal at both time points. Taken together, these confirm that all the constructed pXJ40-Myc vectors containing the inserts of individual SAFV viral protein genes can express the desired proteins in both HEp-2 and Vero cells after transfection.

### 3C PROTEIN IS DEGRADED IN TRANSFECTED CELLS BY THE CELLULAR UBIQUITIN/26S PROTEASOME

**Figure 3:**
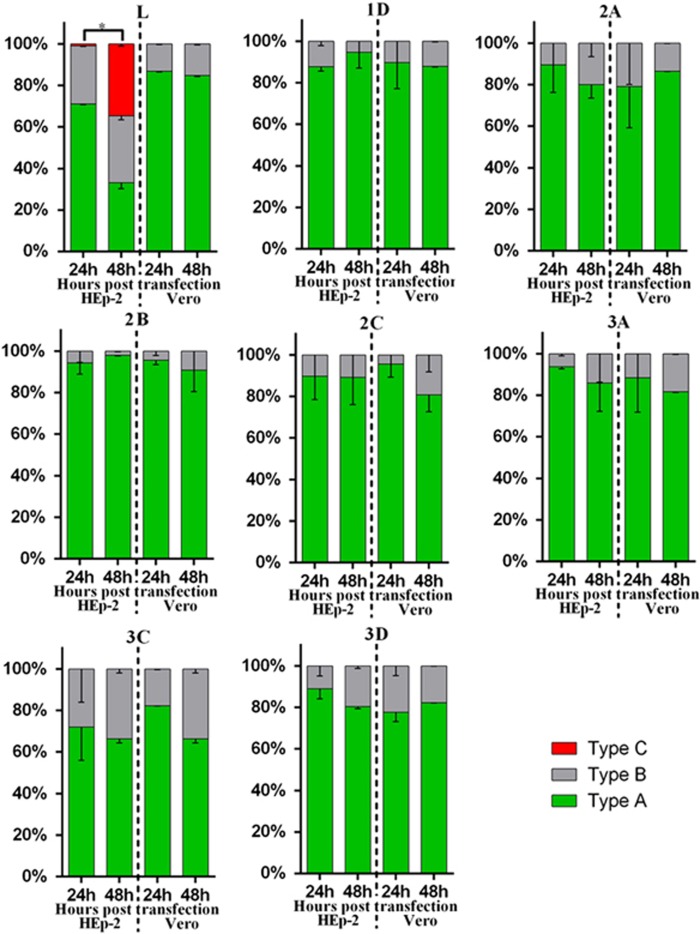
Summary of cellular distributions of L, 1D, 2A, 2B, 2C, 3A, 3C and 3D in transfected HEp-2 and Vero cells. HEp-2 and Vero cells transfected with individual expression plasmids were immunofluorescently stained for various SAFV proteins at 24 and 48 h posttransfection. Intracellular localization of viral proteins was identified based on where the fluorescence signals were observed (types A–C), and the ratio of these types relative to the total number of cells counted was scored. About 100 positive cells from at least five different optical fields were evaluated in each experiment, and results are representative of three independent experiments performed. Chi-square test was used to compare the differences of cellular localization of individual protein between different time points, and differences were considered significant at *P*<0.01 (see Supplementary Table S2 for the complete results of chi-square test). Asterisk, the differences between different time points are significant. Saffold virus, SAFV.

To determine whether the expressed SAFV 3C protein could be similarly degraded early following transfection as seen in the 3C proteins of encephalomyocarditis virus (EMCV) and TMEV,^20, 21, 22^ lysates from HEp-2 and Vero cells transfected with pXJ40-Myc-3C vector were harvested at four-h intervals following transfection and processed for western blotting analysis. In Figures 1C and 3C, it was expressed to a detectable level in HEp-2 cells from four h posttransfection, reached peaks level at 16 h and degraded afterwards and was completely undetectable at 48 h. In Vero cells, 3C was detectable by western blottings from four h posttransfection, reached peak levels at 12 h posttransfection and was totally degraded by 44 h posttransfection (Figure 1E). In addition, transfection of the pXJ40-Myc-3C vector into HEp-2 and Vero cells in the presence of the reversible proteasome inhibitor z-Leu-Leu-Leu CHO (MG132; 20 μM) revealed the stable expression of 3C (Figures 1D and 1F). These confirm that SAFV 3C protein was susceptible to degradation by the cellular ubiquitin/26S proteasome, consistent with previous findings.

### LOCALIZATION OF THE EXPRESSED VIRAL PROTEINS IN THE TRANSFECTED CELLS

To delineate the different types of cellular localization of individual viral protein expressed in HEp-2 or Vero cells, we classified the cells with positive fluorescence signal into three types (Supplementary Figure S1). Type A cells exhibited distinct positive immunofluorescence staining in the cytoplasm only; type B cells exhibited positive staining in the cytoplasm with speckles of fluorescence signals overlapping the nuclear region; type C cells exhibited distinct positive staining in both the cytoplasm and the nucleus. Indirect immunofluorescence assays revealed that both HEp-2 and Vero cells expressing viral 1D, 2A, 2B, 2C, 3A, 3C and 3D proteins could be classified as either type A or type B. As for transfected cells expressing L protein, only HEp-2 cells exhibited immunofluorescent pattern of all three types (types A–C), whereas Vero cells exhibited immunofluorescent pattern of either type A or type B (Figure 2 and Supplementary Figure S2). The majority of HEp-2 and Vero cells expressing viral 1D, 2A, 2B, 2C, 3A, 3C or 3D proteins, as well as Vero cells expressing L protein, exhibited cytoplasmic localization (type A) at both 24 and 48 h posttransfection (Figure 3). Interestingly, the cellular distributions of L protein in transfected HEp-2 cells were distinct between 24 and 48 h (*P*<0.01). HEp-2 cells expressing L protein were mainly of type A category (72%), with limited amounts of type B (28%) and type C (1%) cells at 24 h. At 48 h, transfected HEp-2 cells expressing L protein were evenly distributed into three categories (33% type A, 32% type B and 35% type C). These results suggest the restricted cytoplasmic distribution of 1D, 2A, 2B, 2C, 3A, 3C and 3D proteins. In contrast, the L protein seemed to have been transported from the cytoplasm into the nucleus between 24 and 48 h posttransfection in HEp-2 cells.

### LOCALIZATION OF THE EXPRESSED VIRAL PROTEINS IN INFECTED CELLS CORRELATED THE RESULTS OBTAINED IN THE TRANSFECTED CELLS

**Figure 4:**
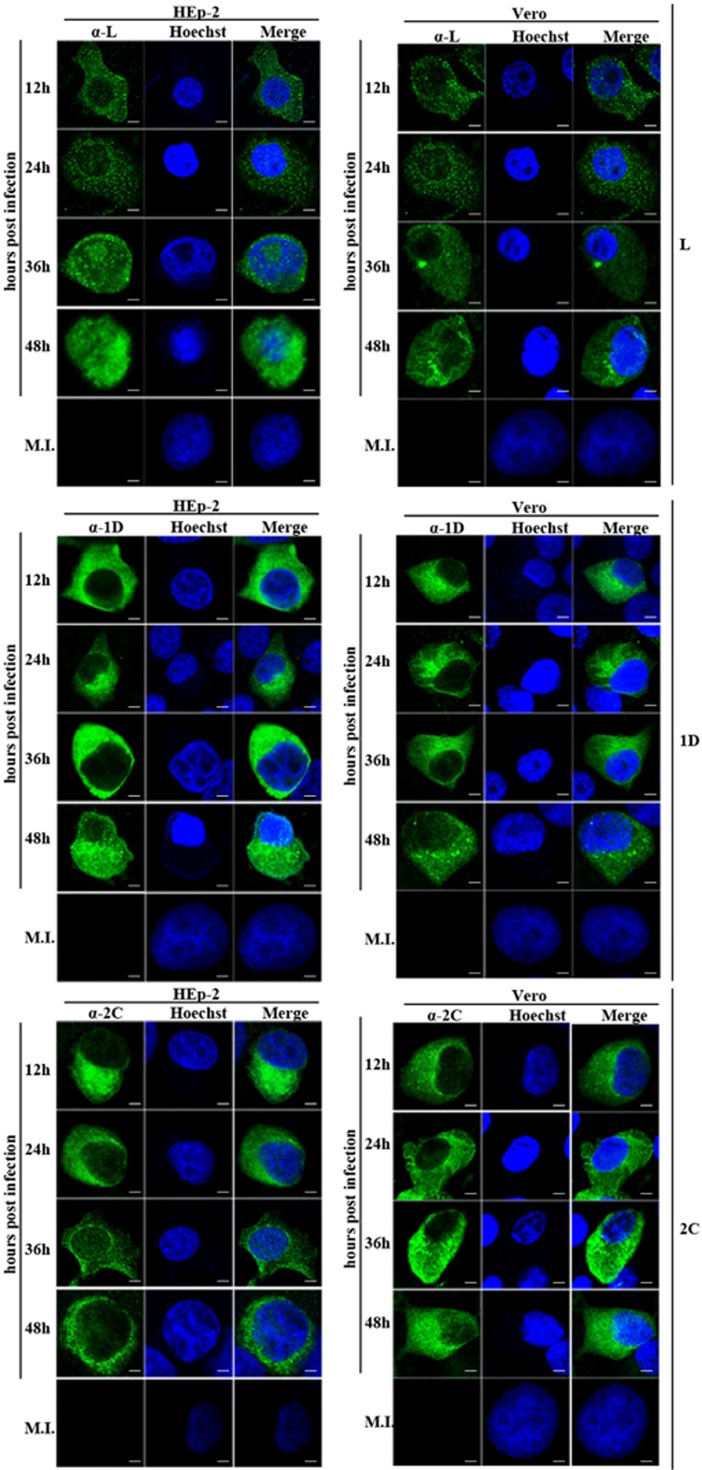
Cellular localization of L, 1D and 2C in infected HEp-2 and Vero cells at various time points. HEp-2 and Vero cells were infected with SAFV at a multiplicity of infection (MOI) of 1.The cells were fixed at 12, 24, 36 or 48 h postinfection and stained with antibody against L, 1D or 2C. Antibody–antigen complexes were detected with swine anti-rabbit immunoglobulins-FITC. Nuclei were stained with Hoechst 33258. Cells were observed with a fluorescence microscope (Leica SP8 laser scanning confocal microscope with a 63 × /1.40 NA oil objective). Merge, merged images of cells FITC-labeled with viral proteins and Hoechst. Mock-infected cells, MI; Saffold virus, SAFV. Scale bar=10 μM.

**Figure 5:**
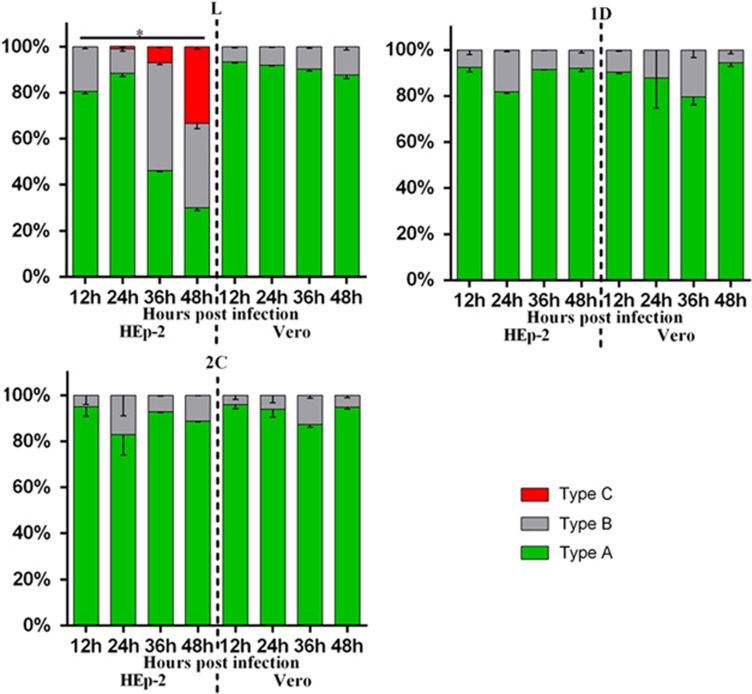
Summary of cellular distributions of L, 1D and 2C in infected HEp-2 and Vero cells. HEp-2 and Vero cells infected with SAFV (MOI=1) were immunofluorescently stained at 12, 24, 36 and 48 h postinfection. Intracellular localization of viral proteins was identified based on where the fluorescence signals were observed (types A–C), and the ratio of these types relative to the total number of cells counted was scored (*n*=100). Results are representative of three independent experiments performed. Chi-square test was used to compare the differences of cellular localization of individual protein between different time points, and differences were considered significant at *P*<0.01 (see Supplementary Table S3 for the complete results of chi-square test). Asterisk, the differences between different time points are significant. Multiplicity of infection, MOI; Saffold virus, SAFV.

To confirm the cellular localization of viral proteins derived from transfection studies, HEp-2 and Vero cells were infected with live SAFV and subsequently analyzed with fluorescence microscopy. Rabbit polyclonal antibodies against L, 1D and 2C proteins were previously generated and used in immunostaining of infected cells—which were fixed every 12 h until 48 h postinfection. We randomly selected approximately 100 cells from at least five distinct optical fields from each group for evaluation. As shown in Figures 4 and 5 and Supplementary Figure S3, SAFV-infected HEp-2 and Vero cells stained with either anti-1D or anti-2C antibody, as well as SAFV-infected Vero cells stained with anti-L antibody, were mainly of type A category with the limited amount of type B cells at all the time points assayed. Similar to the transfected HEp-2 cells expressing L protein, the infected HEp-2 cells stained with anti-L antibody were also represented by all three categories of cells with increasing levels of type C cells from 12 to 48 h postinfection. The percentage of cell types in each category at various time points was also significantly different (*P*<0.01). The findings for L, 1D and 2C protein localization in SAFV-infected Vero and HEp-2 cells corroborate with the results obtained from cellular transfection studies.

### CELL FRACTIONATION FOR L PROTEIN EXPRESSED TRANSFECTED CELLS

**Figure 6:**
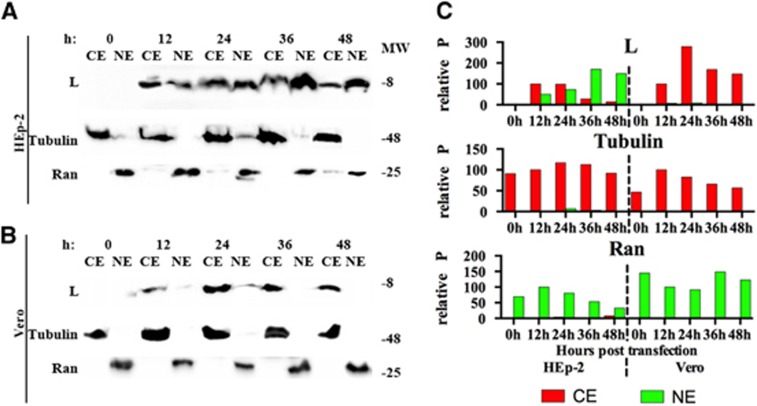
Detection of L proteins in the cellular fractions of transfected cells. Western blottings of (**A**) HEp-2 and Vero (**B**) cells expressing L protein at 12, 24, 36 and 48 h posttransfection with pXJ40-Myc-L. Nuclear and cytoplasmic fractions of HEp-2 and Vero cells were collected every 12 h posttransfection and detected with anti-Myc, anti-β-tubulin and anti-Ran GTPase antibodies. (**C**) The relative P of bands obtained from panels a and b. The ‘relative P' is pixel count (ImageJ software) in the individual protein product, normalized to CE (for L protein and Tubulin) or NE (for Ran GTPase) at 12 h posttransfection. The western blotting results shown here is representative of one of the three repeated experiments. Cytoplasmic extracts, CE; nuclear extracts, NE; molecular weight marker (in thousands Dalton), MW.

To confirm the results obtained with immunofluorescence assay and confocal microscopy, cell fractionation for HEp-2 and Vero cells transfected with L protein was performed at 12, 24, 36 and 48 h posttransfection (Figure 6). In HEp-2 cells, the L protein was detected both in the cytoplasmic and nuclear fractions, and the amount in the nuclear fraction increased temporally (Figure 6A). In Vero cells, the L protein was only detected in the cytoplasmic fractions (Figure 6B). These results confirm the translocation of L protein from the cytoplasm to the nucleus in HEp-2 cells and static cytoplasmic localization in Vero cells.

### THE AMINO ACID AT POSITION 58 IN SER/THR-RICH DOMAIN IS RESPONSIBLE FOR THE NUCLEAR LOCALIZATION OF VIRAL L PROTEIN IN HEP-2 CELLS

**Figure 7:**
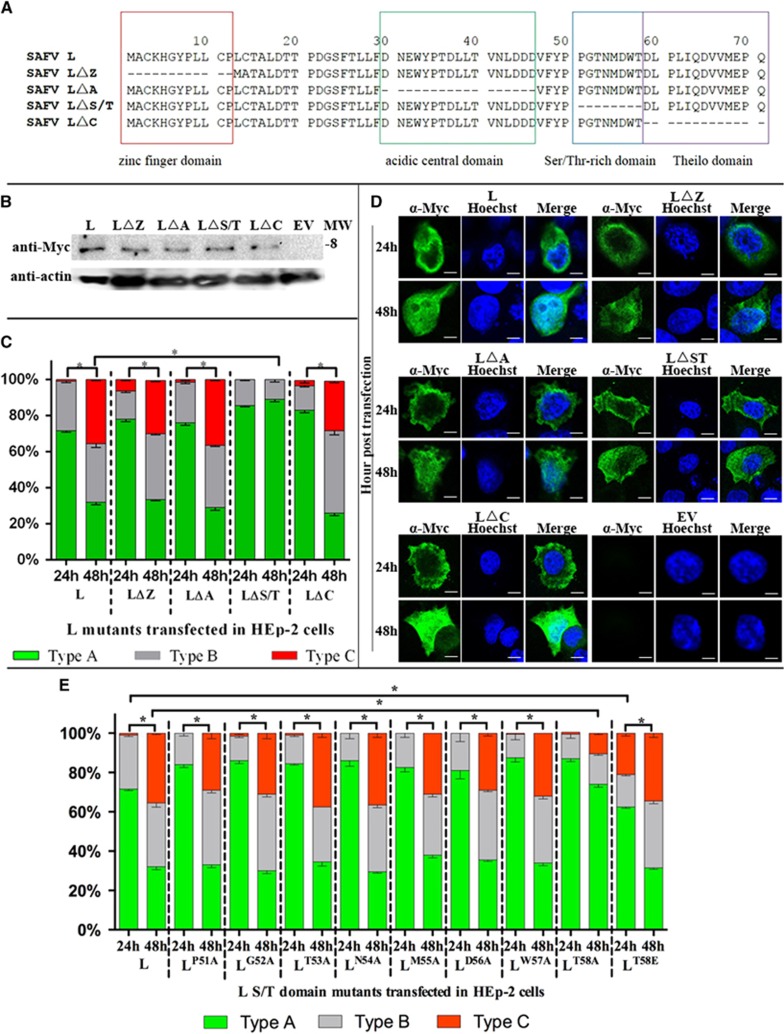
Cellular localization of mutant L protein in transfected HEp-2 cells. (**A**) Amino-acid sequence alignment of SAFV L and mutant L proteins. Zinc finger domain, acidic central domain, Ser/Thr-rich domain and Theilo domain of L protein are highlighted in red, green, blue and purple, respectively. (**B**) Western blottings of the expression of pXJ40-Myc-L and mutated L gene constructs in transfected HEp-2 cells at 24 h posttransfection. The intensity of actin staining served as protein loading control. Western blotting results shown here is representative of one of the three repeated experiments. (**C**) Summary of cellular distributions of L and mutant L proteins in transfected HEp-2 cells at 24 and 48 h posttransfection. Immunofluorescence assay was performed with the HEp-2 cells transfected with L and mutated L expression plasmids. Intracellular localization of viral proteins was identified based on where the fluorescence signals were observed (types A–C), and the ratio of these types relative to the total number of cells counted was scored (*n*=100). Results are representative of three independent experiments performed. Chi-square test was used to compare the differences of cellular localization of individual protein between different time points, and differences were considered significant at *P*<0.01 (see Supplementary Table S4 for the complete results of chi-square test). (**D**) Cellular localization of L and mutant L proteins in transfected HEp-2 cells at 24 and 48 h posttransfection. Samples were processed as described in panel c. Cell nuclei were stained with Hoechst 33258. Cells were observed with a fluorescence microscope (Leica SP8 laser scanning confocal microscope with a 63 × /1.40 NA oil objective). (**E**) Summary of cellular distribution of wild-type L and mutant L proteins in S/T domain in transfected HEp-2 cells at 24 and 48 h posttransfection. Samples were processed as described in panel b (see Supplementary Table S5 for the complete results of chi-square test). Scale bar=10 μM. Asterisk, the differences between different time points are significant. molecular weight marker (in thousands Dalton), MW; merged images of cells stained with anti-Myc and Hoechst, Merge; cells transfected with empty vector, EV.

The L protein of SAFV contains four domains: zinc finger domain, acidic central domain, Ser/Thr-rich domain, and Theilo domain (Figure 7A). To determine which domain is responsible for L protein translocation between cytoplasm and nucleus of HEp-2 cells, we deleted each of these four domains and cloned them into the pXJ40-Myc plasmid to obtain pXJ40-Myc-LΔZ, -LΔA, -LΔS/T and -LΔC. The expression of mutant L proteins in transfected HEp-2 cells was monitored by western blotting assay at 24 h posttransfection. The Myc-tagged mutant L proteins were detected in the expected molecular sizes (Figure 7B). Indirect immunofluorescence staining of transfected HEp-2 cells revealed the different cellular distribution of LΔZ, LΔA and LΔC proteins between 24 and 48 h posttransfection (*P*<0.01), and the distribution patterns were similar to wild-type L protein (*P*>0.01) (Figures 7C and 7D). In contrast, the cellular localization of LΔS/T protein exhibited similar patterns between 24 and 48 h posttransfection in HEp-2 cells (*P*>0.01), but the distribution pattern was determined to be significantly different compared with wild-type L protein (*P*<0.01). These results suggest the importance of Ser/Thr-rich domain for the nuclear translocation of L protein in HEp-2 cells.

To further determine the essential residues in the nuclear translocation of L protein in HEp-2 cells, Alanine-scanning mutagenesis of the Ser/Thr-rich domain was performed (Figure 7E). Among the derived mutants, only L^T58A^ exhibited a distribution pattern in HEp-2 cells that was significantly different from wild-type L protein. The distribution pattern of L^T58A^ is comparable to that of LΔS/T. This result suggests that Thr in position 58 of L protein is the crucial residue for the nuclear translocation of L protein in HEp-2 cells.

As Thr 58 is predicted to be a possible phosphorylation site in SAFV L, we mimicked phosphothreonine and created a construct expressing L^T58E^ (Figure 7E). The L^T58E^ was localized mainly in the cytoplasm (type A) at 24 h posttransfection and both the cytoplasm and nucleus (evenly distributed as types A–C) at 48 h posttransfection. Although the L^T58E^ trends to distribute to the nucleus (type B and C) comparing to the wild-type L at 24 h posttransfection (*P*<0.01), the cellular distribution pattern of the L^T58E^ is similar to the wild-type L instead of L^T58A^. This finding indicates that the phosphorylation of Thr 58 might have a key role in the nuclear localization of L protein in HEp-2 cells.

## Discussion

Although essential infection activities of picornaviruses are believed to take place in the cytoplasm, several viral proteins—such as 2A and 3BCD of EMCV—are also known to be translocated to the nucleus and interfere with transcription or translation of cellular proteins.^23, 24^ This study demonstrates that most of the viral proteins of SAFV are localized in the cytoplasm in both HEp-2 and Vero cells. In contrast, L was translocated from the cytoplasm into the nucleus of HEp-2 cells but remained in the cytoplasm of Vero cells during the infection process. The cellular localization of L protein in HEp-2 cells is also unique and was not observed in other mammalian cell lines (NIH/3T3, CHO-K1 and Neuro2A cells) at 48 h posttransfection (Supplementary Figure S4). Nuclear localization signals are absent in the SAFV L protein sequence, suggesting that, in order to move between different cellular compartments, the L protein may have additional unidentified interacting cellular protein partners. These proteins may be either present only in HEp-2 cells but not in other cells or present in other cell lines but in a form that is unable to interact with SAFV-L. These cellular proteins may be transported into the nucleus or interact with nucleoporins during SAFV infection and, consequently, transport the L protein from the cytoplasm to the nucleus. The possible interaction between L protein (or its interacting cellular partners) and nucleoporins is supported by a recently published report that L protein of SAFV induces Phe/Gly-containing nucleoporins to undergo phosphorylation.^10^ The phosphorylation of nucleoporins by L protein requires the phosphorylation of L protein in advance.^25^ Furthermore, our study demonstrates that the phosphorylation of Thr 58 in the Ser/Thr-rich domain of L protein has a critical role in the cellular distribution of SAFV L in HEp-2 cells, which also suggests the possible role of L protein in the phosphorylation of nucleoporins. It may be possible SAFV L protein interacts with a currently unidentified cellular protein through Thr 58, and our future work will focus on identifying the cellular proteins that interact with L protein in HEp-2 and Vero cells during SAFV infection and determining which of these are crucial to the protein's nuclear localization. It would also be of interest to elucidate the role of SAFV L in the nucleocytoplasmic trafficking of cellular proteins.

The differential distributions of L protein in Vero cells and HEp-2 cells during the late stage of SAFV infection may have led to variations in the functions of L protein during SAFV infection in these two types of cell lines. Based on our previous work, SAFV induces apoptosis in HEp-2, NIH/3T3, CHO-K1 and Neuro2A cells, but the process of apoptosis fails to proceed to the end stage in HEp-2 cells.^16^ The mechanism of this difference in cellular responses to SAFV infection between HEp-2 and other cell lines has not yet been elucidated. One possible explanation is that the cytoplasmic-nuclear translocation of L protein in HEp-2 may disrupt the production of certain protein(s) required to complete the apoptotic cascade. This hypothesis needs to be confirmed by further investigation.

In our experiment, 3C protein was degraded in HEp-2 cells from 16 h after transfection and Vero cells from 12 h after transfection but was stabilized in the presence of MG132. The results were not unexpected in light of the reported rapid degradation *in vitro* and *in vivo* of the related cardiovirus TMEV and EMCV 3C proteins.^20, 21, 22^ The mechanism of TMEV and EMCV 3C degradation has been well studied both *in vitro* and *in vivo*. TMEV 3C and EMCV 3C are degraded by the ubiquitin/26S proteasome, and a 10 amino-acid sequence, LLLRAHLFVV in TMEV 3C and LLVRGRTLVV in EMCV 3C, near the N-terminus was identified as the signal targeted for ubiquitin/26S proteasomal degradation.^20, 22, 26^ The instability of SAFV 3C may be due to the presence of a 10 amino-acid sequence, LLIKGHLFVV (Supplementary Figure S5) that could target the protein for degradation. At 48 h after transfection in both HEp-2 and Vero cells, 3C was undetectable by western blotting assay, whereas still detectable in the cytoplasm of <1% of the total number of cells by immunofluorescence assay. The small number of positive cells may have accounted for the failure of detection of 3C protein by western blottings.

In conclusion, most of the SAFV viral proteins investigated in this study localize in the cytoplasm of both HEp-2 and Vero cells at various time points. The L protein is uniquely localized in the nucleus and cytoplasm of HEp-2 cells at the late stage of infection (36 h postinfection) or transfection (48 h posttransfection); the Thr in position 58 of L protein is responsible for this unique cellular localization in HEp-2 cells. The viral 3C protein is degraded from the early phase of transfection by the cellular ubiquitin/26S proteasome. The findings may provide some clues to the mechanism of different cellular responses of SAFV-infected HEp-2 cells in comparison with other SAFV-infected mammalian cell lines and allude to the crucial role of L protein in the process of virus infection.
