# Supplementary material for: Intracellular localization of Saffold virus Leader (L) protein differs in Vero and HEp-2 cells
Source: Emerg Microbes Infect. 2016 Oct 12;5(10):e109–. doi: 10.1038/emi.2016.110 (PMC5117731; doi:10.1038/emi.2016.110)
Supplement: Supplementary Information [file emi2016110x1.pdf]

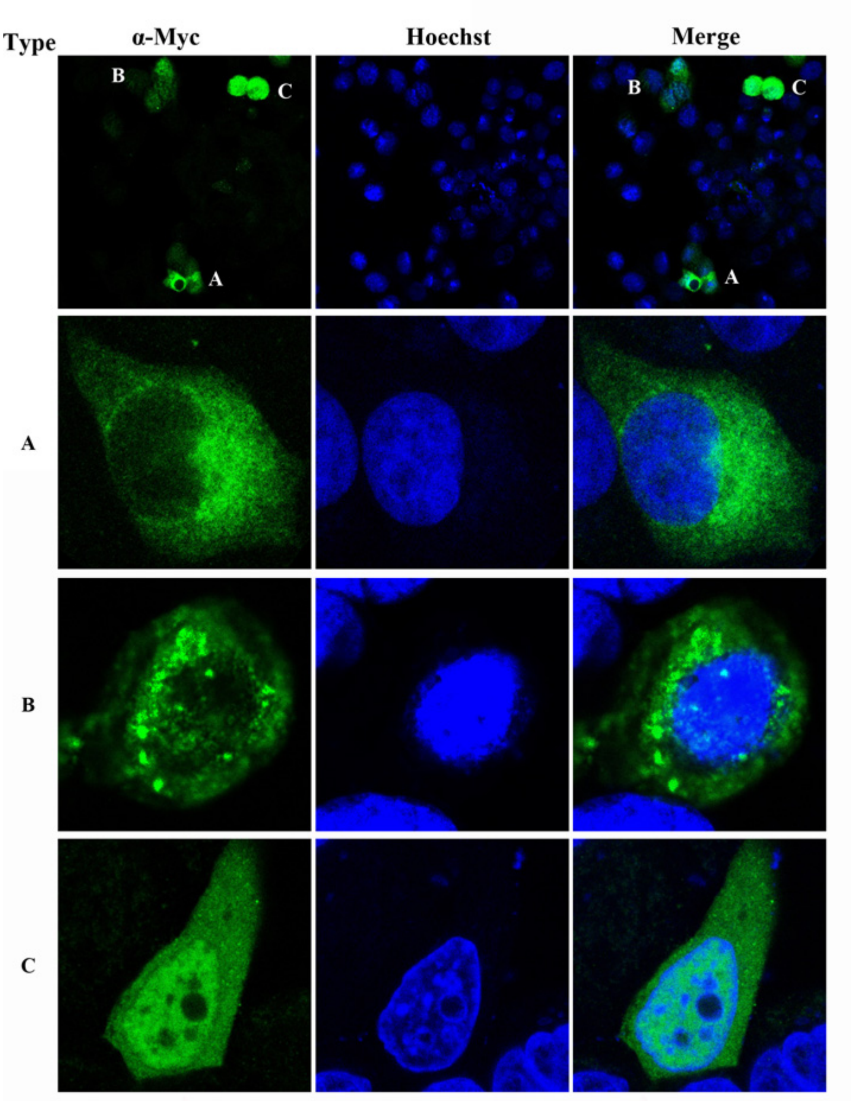

Supplemantary Figure S1 Characterization of transfected or infected cells with the cellular localization of fluorescence signals. HEp-2 cells transfected with pXJ40-MyC-L were stained with the antibody against Myc at 48 hours post transfection. Nuclei were stained with Hoechst 33258 (Hoechst). Cells were observed with a fluorescence microscope (Leica SP8 laser scanning confocal microscope). Merge represents merged images stained with anti-Myc and Hoechst. Type A cells had clear-cut positive immunofluorescence staining in the cytoplasm. Type B cells had positive staining in the cytoplasm with speckles of positive staining overlapping onto nuclear region, and Type C cells had clear-cut positive staining in both the cytoplasm and nucleus.
