# Supplementary material for: Intracellular localization of Saffold virus Leader (L) protein differs in Vero and HEp-2 cells
Source: Emerg Microbes Infect. 2016 Oct 12;5(10):e109–. doi: 10.1038/emi.2016.110 (PMC5117731; doi:10.1038/emi.2016.110)
Supplement: Supplementary Information [file emi2016110x2.pdf]

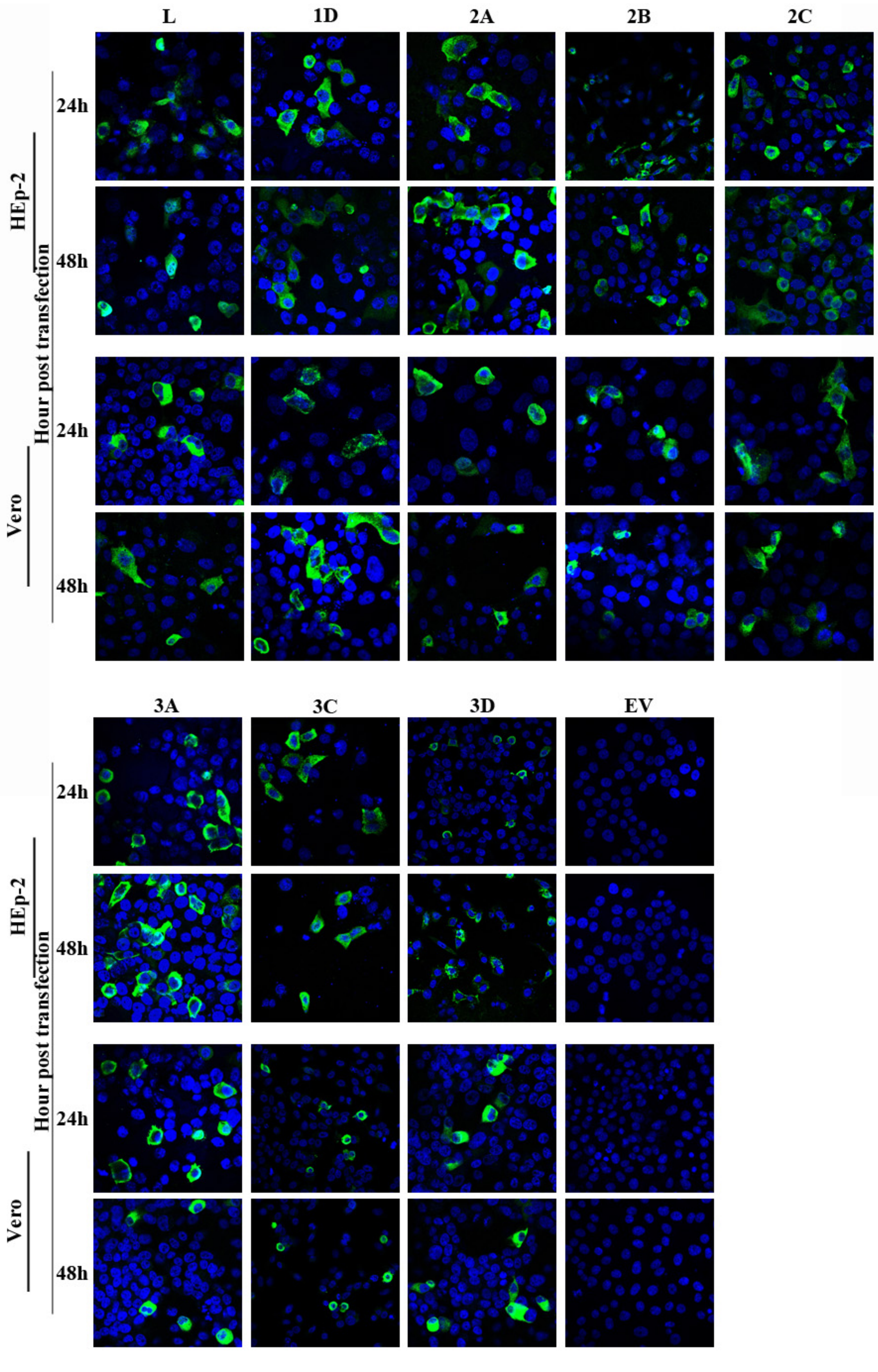
L
1D
2A
2B
2C
HEp-2
Vero
Hour post transfection
24h
48h
24h
48h
3A
3C
3D
EV
HEp-2
Vero
Hour post transfection
24h
48h
24h
48h

Supplementary Figure S2 Cellular localization of viral proteins in transfected cells (large field of view). Immunofluorescent detection of individual SAFV proteins—L, 1D, 2A, 2B, 2C, 3A, 3C, and 3D—in transfected HEp-2 and Vero cells at 24h and 48h post-transfection. HEp-2 and Vero cells were transfected with the expression plasmids pXJ40-Myc-SAFV virus gene constructs, fixed at 24h or 48h post-transfection, and immunofluorescently stained with the anti-Myc antibody (Green). Cell nuclei were stained with Hoechst 33258 (blue). Cells were observed with a fluorescence microscope (Leica SP8 laser scanning confocal microscope with a 40×/1.30 NA oil objective). EV represents cells transfected with empty vector. Magnification: X400.
