# Supplementary material for: Intracellular localization of Saffold virus Leader (L) protein differs in Vero and HEp-2 cells
Source: Emerg Microbes Infect. 2016 Oct 12;5(10):e109–. doi: 10.1038/emi.2016.110 (PMC5117731; doi:10.1038/emi.2016.110)
Supplement: Supplementary Information [file emi2016110x3.pdf]

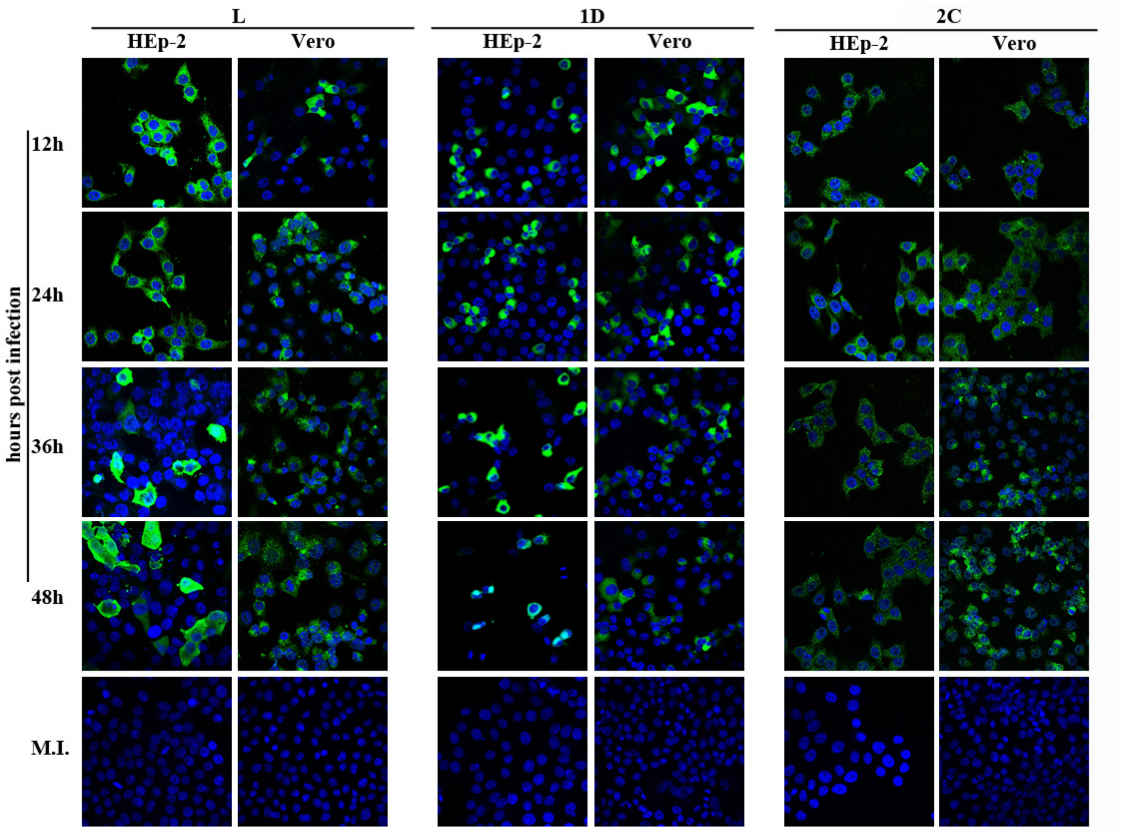

Supplementary Figure S3 Cellular localization of L, 1D, and 2C in infected HEp-2 and Vero cells at various time points (large field of view). HEp-2 and Vero cells were infected with SAFV at a multiplicity of infection (MOI) of 1.The cells were fixed at 12h, 24h, 36h or 48h post-infection and stained with antibody against L, 1D, or 2C. Antibody-antigen complexes were detected with swine anti-rabbit immunoglobulins-FITC (Green). Nuclei were stained with Hoechst 33258 (Blue). Cells were observed with a fluorescence microscope (Leica SP8 laser scanning confocal microscope with a 40×/1.30 NA oil objective). *M.I.*, mock-infected cells. Magnification: X400.
