# Supplementary material for: Intracellular localization of Saffold virus Leader (L) protein differs in Vero and HEp-2 cells
Source: Emerg Microbes Infect. 2016 Oct 12;5(10):e109–. doi: 10.1038/emi.2016.110 (PMC5117731; doi:10.1038/emi.2016.110)
Supplement: Supplementary Information [file emi2016110x4.pdf]

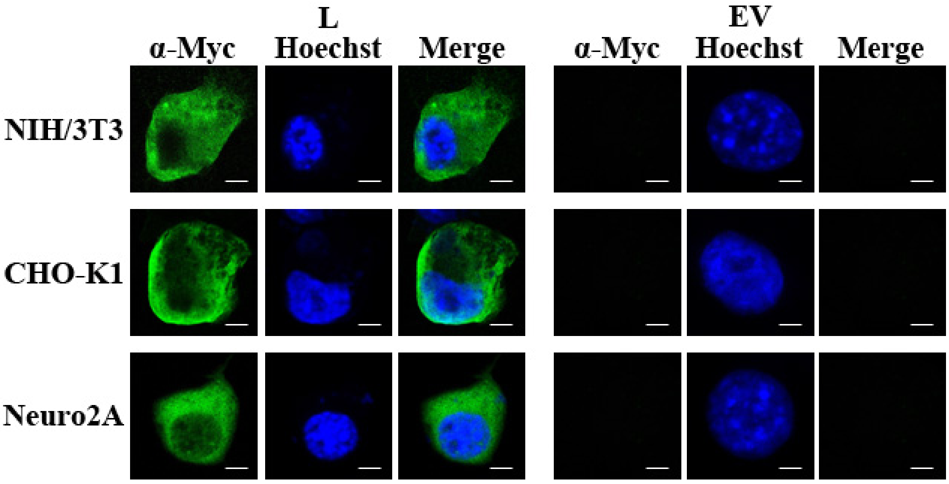

Supplementary Figure S4 Cellular localization of L protein in transfected NIH/3T3, CHO-K1, and Neuro2A cells at 48 hours post-transfection. NIH/3T3, CHO-K1, and Neuro2A cells were transfected with the expression plasmids pXJ40-Myc-L or pXJ40-Myc, fixed at 48 hours post-transfection and stained with the anti-Myc antibody for L. Cell nuclei were stained with Hoechst 33258. Cells were observed with a fluorescence microscope (Leica SP8 laser scanning confocal microscope with a 63×/1.40 NA oil objective). Merge represents the merged images stained with anti-Myc and Hoechst. EV represents cells transfected with empty vector pXJ40-MyC. Scale bar = 10 μm.
