# Supplementary material for: Intracellular localization of Saffold virus Leader (L) protein differs in Vero and HEp-2 cells
Source: Emerg Microbes Infect. 2016 Oct 12;5(10):e109–. doi: 10.1038/emi.2016.110 (PMC5117731; doi:10.1038/emi.2016.110)
Supplement: Supplementary Information [file emi2016110x5.pdf]

```
                                                      ubiquitin/26S proteasomal degradation signal
                                                                   ↓
                        10         20         30         40         50         60         70         80
EMCV            --------GP NPVMDFEKYV AKHVTAPIGF VYP-TGVSTQ TCLLVRGRTL VVNRHMAESD WTSIVVRGVT HARSTVKILA
TMEV BeAn 8386  GGGKVLAQAG NPVMDFELFC AKNIVAPITF YYPDKAEVTQ SCLLLRAHLF VVNRHVAETD WTAFKLKDVR HERHTVALRS
SAFV-Panang     GGGKIVAQAG NPVMDYEVNI AKNMVTPITF FYADKAQVTQ SCLLIKGHLF VVNRHVAETD WCAFELKGIR HERDSVQMRS

                        90        100        110        120        130        140        150        160
EMCV            IAKAGKETDV SFIRLSSGPL FRDNTSKFVK AGDVLPTGAA PVTGIMNTDI PMMYTGTFLK AGVSVPVETG QTFNHCIHYK
TMEV BeAn 8386  VNRSGAKTDL TFIKVTKGPL FKDNVNKFCS NKDDFPARND TVTGIMNTGL AFVYSGNFLI GNQPVNTTTG ACFNHCLHYR
SAFV-Panang     VNKSGMEVDL TFVKVVKGPL FKDNSKKFCS KDDDFPARNE TVTGIMNTGV PFVFTGKFLI GNQPVNTTTG ACFNHCIHYR

                       170        180        190        200        210
EMCV            ANTRKGWCGS ALLADLGGSK KILGIHSAGS MGIAAASIVS QEMIRAVVN- --AFEPQ
TMEV BeAn 8386  AQTRRGWCGS AIICNVNGKK AVYGMHSAGG GGLAAATIIT KELIEAAEKS MLALEPQ
SAFV-Panang     ATTHRGWCGS ALICHVNGKK AVYAMHSAGG GGMAAATIVT QEMIEAAEKA LDCLTPQ
```

Supplementary Figure S5 Amino acid sequence alignment of cardioviruses 3C proteins. The 3C amino acid sequence of Encephalomyocarditis virus (EMCV, Genbank number: NC_001479), Theiler's murine encephalomyelitis virus (strain BeAn 8386) (TMEV, Genbank number: M16020), and Saffold virus Penang strain (SAFV, Genbank number: HQ162476). The domain that serves as a signal of ubiquitin/26S proteasomal degradation is highlighted in yellow.
