# Supplementary material for: Intracellular localization of Saffold virus Leader (L) protein differs in Vero and HEp-2 cells
Source: Emerg Microbes Infect. 2016 Oct 12;5(10):e109–. doi: 10.1038/emi.2016.110 (PMC5117731; doi:10.1038/emi.2016.110)
Supplement: Supplementary Information [file emi2016110x6.pdf]

Supplementary Table S1 Primers used in this study

Name	Sequence (5'-3')	Outcome Plasmid
L-XhoI-Fwd[a]	CAG ACT CGA GAT GGC GTG CAA ACA CGG	pXJ40-Myc-L<br>pXJ40-MyC-L∆ST<br>pXJ40-MyC-L∆C
L-PstI-Rev[b]	CAG ACT GCA GTT GCG GTT CCA TCA CAA CAT CC	pXJ40-Myc-L;<br>pXJ40-Myc-L∆Z
1D-BamHI-Fwd	CAG AGG ATC CGG TGT AGA TAA TGC TG	pXJ40-Myc-1D
1D-PstI-Rev	CAG ACT GCA GTT GCA ATT CTA GAG TAG GAG TCG	
2A-BamHI-Fwd	CAG AGG ATC CAA TCC AAT TTC AAT TTA CAG AGT TGA TTT GTT CAT AAA CTT C	pXJ40-Myc-2A
2A-XhoI-Rev	CAG ACT CGA GTT GCA ACT GAA AAA CCG ACT GAA CGG	
2B-BamHI-Fwd	CAG AGG ATC CGG TGG GGT GCT AAC TAA ATC C	pXJ40-Myc-2B
2B-PstI-Rev	CAG ACT GCA GTT GTT GTT GGA GAA GCG G	
2C-XhoI-Fwd	CAG ACT CGA GTC TCC TAT TAG AGA AGC CAA TGA TAG	pXJ40-Myc-2C
2C-PstI-Rev	CAG ACT GCA GTT GAG CGA CCA ATG TGT TC	
3A-BamHI-Fwd	CAG AGG ATC CTC ACC TGG TAA TGA CAT GG	pXJ40-Myc-3A
3A-XhoI-Rev	CAG ACT CGA GTT GTT CTC CTT CAG AC	
3C-BamHI-Fwd	CAG AGG ATC CGG TGG AGG AAA GAT TGT AGC CC	pXJ40-Myc-3C
3C-XhoI-Rev	CAG ACT CGA GTT GAG GGG TTA AAC AAT CTA AAG CTT TTT CAG CAG	
3D-XhoI-Fwd	CAG ACT CGA GGG AGC TAT AGT TGA AAT TGG CAT C	pXJ40-Myc-3D
3D-PstI-Rev	CAG ACT GCA GTC TAA ATA GAT TGA GCC ATC TAT AGA GCA TGG	
L∆Z-Xho1-Fwd	CAGACTCGAGATGGCGTGCTTTGGATACTACTCCAGACGGATC	pXJ40-Myc-L∆Z
L∆A-Fwd-Xho1-Pst1	TCGAGATGGCGTGCAAACACGGATATCCGCTTTTGTGCCCTCTTTGCACTGCTTTGGATACTACTCCAGACGGATCTTTCACTCTCTTGTTTGTGTTTTATCCTCCTGGTACCAACATGGACTGGACCGATTTGCCATTGATTCAGGATGTTGTGATGGAACCGCAACTGCA	pXJ40-MyC-L∆A
L∆A-Rev-Xho1-Pst1	GTTGCGGTTCCATCACAACATCCTGAATCAATGGCAAATCGGTCCAGTCCATGTTGGTACCAGGAGGATAAAACACAAACAAGAGAGTGAAAGATCCGTCTGGAGTAGTATCCAAAGCAGTGCAAAGAGGGCACAAAAGCGGATATCCGTGTTTGCACGCCATC	pXJ40-MyC-L∆A
L∆S/T-Rev1	CAAATCAGGATAAAACACGTCGTCGTCC	pXJ40-MyC-L∆ST
L∆S/T-PstI-Rev2	CAGACTGCAGTTGCGGTTCCATCACAACATCCTGAATCAATGGCAAATCAGGATAAAAC	pXJ40-MyC-L∆ST
L∆C-Pst1-Rev	CAGACTGCAGGGTCCAGTCCATGTTGGTACCAGGAGG	pXJ40-MyC-L∆C
L^{P51A}-Fwd	GACGTGTTTTATCCTGCTGGTACCAACATG	pXJ40-MyC-L^{P51A}
L^{P51A}-Rev	CATGTTGGTACCAGCAGGATAAAACACGTC	
L^{G52A}-Fwd	GTTTTATCCTCCTGCTACCAACATGGACTG	pXJ40-MyC-L^{G52A}
L^{G52A}-Rev	CAGTCCATGTTGGTAGCAGGAGGATAAAAC	

L^{T53A}-Fwd	GTTTTATCCTCCTGGTGCCAACATGGACTGGAC	pXJ40-MyC-L^{T53A}
L^{T53A}-Rev	GTCCAGTCCATGTTGGCACCAGGAGGATAAAAC	
L^{N54A}-Fwd	GTTTTATCCTCCTGGTACCGCAATGGACTGGACCGATTTG	pXJ40-MyC-L^{N54A}
L^{N54A}-Rev	CAAATCGGTCCAGTCCATTGCGGTACCAGGAGGATAAAAC	
L^{M55A}-Fwd	CTCCTGGTACCAACGCCGACTGGACCGATTTG	pXJ40-MyC-L^{M55A}
L^{M55A}-Rev	CAAATCGGTCCAGTCGGCGTTGGTACCAGGAG	
L^{D56A}-Fwd	GGTACCAACATGGCCTGGACCGATTTG	pXJ40-MyC-L^{D56A}
L^{D56A}-Rev	CAAATCGGTCCAGGCCATGTTGGTACC	
L^{W57A}-Fwd	GGTACCAACATGGACGCGACCGATTTGCCATTG	pXJ40-MyC-L^{W57A}
L^{W57A}-Rev	CAATGGCAAATCGGTCGCGTCCATGTTGGTACC	
L^{T58A}-Fwd	CAACATGGACTGGGCCGATTTGCCATTG	pXJ40-MyC-L^{T58A}
L^{T58A}-Rev	CAATGGCAAATCGGCCCAGTCCATGTTG	
L^{T58E}-Fwd	GTACCAACATGGACTGGGAGGATTTGCCATTGATTCAG	pXJ40-MyC-L^{T58E}
L^{T58E}-Rev	CTGAATCAATGGCAAATCCTCCCAGTCCATGTTGGTAC	
L-SacI-Fwd	CAG AGA GCT CAT GGC GTG CAA ACA CGG	pQE30-L
L-SacI-Rev	CAG AGA GCT CTT GCG GTT CCA TCA CAA CAT CC	
1D-SacI-Fwd	CAG AGA GCT CGG TGT AGA TAA TGC TG	pQE30-1D
1D-KpnI-Rev	CAG AGG TAC CTT GCA ATT CTA GAG TAG GAG TCG	
2C-SacI-Fwd	CAG AGA GCT CTC TCC TAT TAG AGA AGC CAA TGA TAG	pQE30-2C
2C-SacI-Rev	CAG AGA GCT CTT GAG CGA CCA ATG TGT TC	

[a]Fwd, Forward Primer. [b]Rev, Reverse primer.
