# Supplementary material for: Intracellular localization of Saffold virus Leader (L) protein differs in Vero and HEp-2 cells
Source: Emerg Microbes Infect. 2016 Oct 12;5(10):e109–. doi: 10.1038/emi.2016.110 (PMC5117731; doi:10.1038/emi.2016.110)
Supplement: Supplementary Information [file emi2016110x7.pdf]

**Supplementary Table S2** The percentage of immunofluorescent positive Type A, B and C transfected HEp-2 and Vero cells at 24 and 48 hours post-transfection. Chi-square test was used to assess the statistical significant of differences for cellular localization of individual viral protein in transfected HEp-2 and Vero cells at 24 and 48 hours post-transfection

| | | **24h post-transfection** | | | **48h post-transfection** | | | ***p*-value** |
|---|---|---|---|---|---|---|---|---|
| | | **A*** | **B** | **C** | **A** | **B** | **C** | |
| HEp-2 | L | 71% | 28% | 1% | 33% | 32% | 35% | <0.001 |
| | 1D | 88% | 12% | 0% | 95% | 5% | 0% | 0.126 |
| | 2A | 89% | 11% | 0% | 80% | 20% | 0% | 0.117 |
| | 2B | 94% | 6% | 0% | 98% | 2% | 0% | 0.279 |
| | 2C | 90% | 10% | 0% | 89% | 11% | 0% | 1.000 |
| | 3A | 94% | 6% | 0% | 86% | 14% | 0% | 0.097 |
| | 3C | 72% | 28% | 0% | 66% | 34% | 0% | 0.445 |
| | 3D | 89% | 11% | 0% | 80% | 20% | 0% | 0.117 |
| Vero | L | 87% | 13% | 0% | 85% | 15% | 0% | 0.839 |
| | 1D | 90% | 10% | 0% | 88% | 12% | 0% | 0.822 |
| | 2A | 79% | 21% | 0% | 86% | 14% | 0% | 0.264 |
| | 2B | 96% | 4% | 0% | 91% | 9% | 0% | 0.251 |
| | 2C | 96% | 4% | 0% | 81% | 19% | 0% | 0.040 |
| | 3A | 88% | 12% | 0% | 82% | 18% | 0% | 0.322 |
| | 3C | 82% | 18% | 0% | 66% | 34% | 0% | 0.015 |
| | 3D | 78% | 22% | 0% | 82% | 18% | 0% | 0.596 |

*A, Type A. B, Type B. C, Type C.
