# Supplementary material for: Intracellular localization of Saffold virus Leader (L) protein differs in Vero and HEp-2 cells
Source: Emerg Microbes Infect. 2016 Oct 12;5(10):e109–. doi: 10.1038/emi.2016.110 (PMC5117731; doi:10.1038/emi.2016.110)
Supplement: Supplementary Information [file emi2016110x8.pdf]

Supplementary Table S3 The percentage of immunofluorescent positive Type A, B and C SAFV-infected HEp-2 and Vero cells at various time points post-fection. Chi-square test was used to assess the statistical significant of differences for cellular localization of L, 1D, or 2C in infected HEp-2 and Vero cells at 12, 24, 36, and 48 hours post-infection

		**12hpi***			**24hpi**			**36hpi**			**48hpi**			***p*-value**
		**A****	**B**	**C**	**A**	**B**	**C**	**A**	**B**	**C**	**A**	**B**	**C**	
HEp-2	L	80%	20%	0%	88%	10%	1%	46%	47%	7%	30%	37%	33%	<0.001
	1D	93%	7%	0%	82%	18%	0%	92%	8%	0%	92%	8%	0%	0.051
	2C	95%	5%	0%	83%	17%	0%	93%	7%	0%	89%	11%	0%	0.032
Vero	L	93%	7%	0%	92%	8%	0%	90%	10%	0%	88%	12%	0%	0.662
	1D	90%	10%	0%	88%	12%	0%	80%	20%	0%	94%	6%	0%	0.025
	2C	96%	4%	0%	94%	6%	0%	87%	13%	0%	95%	5%	0%	0.080

*hpi, hours post infection. ** A, Type A. B, Type B. C, Type C.
