# Supplementary material for: Intracellular localization of Saffold virus Leader (L) protein differs in Vero and HEp-2 cells
Source: Emerg Microbes Infect. 2016 Oct 12;5(10):e109–. doi: 10.1038/emi.2016.110 (PMC5117731; doi:10.1038/emi.2016.110)
Supplement: Supplementary Information [file emi2016110x9.pdf]

Supplementary Table S4 The percentage of immunofluorescent positive Type A, B and C transfected HEp-2 at 24 and 48 hours post-transfection. Chi-square test was used to assess the statistical significant of differences for cellular localization of L protein and mutated L protein in transfected HEp-2 at 24 and 48 hours post-transfection.

		**24h post-transfection**			**48h post-transfection**			***p*-value**
		**A***	**B**	**C**	**A**	**B**	**C**	
HEp-2	L	71%	28%	1%	33%	32%	35%	<0.001
	LΔZ	77%	16%	7%	33%	36%	30%	<0.001
	***p*-value (L& LΔZ)**	0.018			0.735			
	LΔA	77%	22%	1%	28%	35%	37%	<0.001
	***p*-value (L& LΔA)**	0.018			0.741			
	LΔST	86%	14%	0%	90%	10%	0%	0.384
	***p*-value (L& LΔST)**	0.029			<0.001			
	LΔC	84%	13%	2%	25%	47%	27%	<0.001
	***p*-value (L& LΔC)**	0.027			0.083			

*A, Type A. B, Type B. C, Type C.
