# Supplementary material for: Intracellular localization of Saffold virus Leader (L) protein differs in Vero and HEp-2 cells
Source: Emerg Microbes Infect. 2016 Oct 12;5(10):e109–. doi: 10.1038/emi.2016.110 (PMC5117731; doi:10.1038/emi.2016.110)
Supplement: Supplementary Information [file emi2016110x10.pdf]

Supplementary Table S5 The percentage of immunofluorescent positive Type A, B and C transfected HEp-2 at 24 and 48 hours post-transfection. Chi-square test was used to assess the statistical significant of differences for cellular localization of L protein and mutated L protein in transfected HEp-2 at 24 and 48 hours post-transfection.

		**24h post-transfection**			**48h post-transfection**			***p*-value**
		**A***	**B**	**C**	**A**	**B**	**C**	
HEp-2	L	71%	28%	1%	33%	32%	35%	<0.001
	$L^{P51A\,\#}$	84%	16%	0%	33%	38%	29%	<0.001
	***p*-value (L & L^{P51A})**	0.068			0.584			
	L^{G52A}	86%	12%	2%	30%	39%	31%	<0.001
	***p*-value (L & L^{G52A})**	0.017			0.584			
	L^{T53A}	85%	14%	1%	35%	28%	37%	<0.001
	***p*-value (L & L^{T53A})**	0.052			0.827			
	L^{N54A}	86%	14%	0%	30%	34%	36%	<0.001
	***p*-value (L & L^{N54A})**	0.029			0.897			
	L^{M55A}	83%	17%	0%	38%	31%	31%	<0.001
	***p*-value (L & L^{M55A})**	0.099			0.737			
	L^{D56A}	81%	19%	0%	36%	35%	29%	<0.001
	***p*-value (L & L^{D56A})**	0.184			0.661			
	L^{W57A}	87%	12%	1%	34%	34%	32%	<0.001
	***p*-value (L & L^{W57A})**	0.018			0.900			
	L^{T58A}	87%	12%	1%	74%	16%	10%	0.011
	***p*-value (L & L^{T58A})**	0.018			<0.001			
	L^{T58E}	63%	17%	20%	31%	33%	36%	<0.001
	***p*-value (L & L^{T58E})**	<0.001			0.955			

*A, Type A. B, Type B. C, Type C.

$^{\#}L^{P51A}$, mutated L protein with amino acid P in position 51 changed into A.
